# Transcriptomic profiling identifies novel mechanisms of transcriptional regulation of the cytochrome P450 (*Cyp*)*3a11* gene

**DOI:** 10.1038/s41598-019-43248-w

**Published:** 2019-04-30

**Authors:** Guncha Taneja, Suman Maity, Weiwu Jiang, Bhagavatula Moorthy, Cristian Coarfa, Romi Ghose

**Affiliations:** 1 Department of Pharmacological and Pharmaceutical Sciences, University of Houston, 4849 Calhoun Rd., Houston, TX 77204 USA; 2 Advanced Technology Cores, Baylor College of Medicine, One Baylor Plaza, Houston, TX 77030 USA; 3 Department of Pediatrics, Section of Neonatology, Texas Children’s Hospital, Baylor College of Medicine, 1102 Bates Avenue, Suite 530, Houston, TX 77030 USA; 4 Dan L Duncan Comprehensive Cancer Center, Center for Precision Environmental Health, Molecular and Cellular Biology Department, Baylor College of Medicine, One Baylor Plaza, Houston, TX 77030 USA; 5 Present Address: DILIsym Services, A Simulations Plus Company, Research Triangle Park, North Carolina, 27709 USA

**Keywords:** Drug regulation, Toxicology, Toxicology, Toxicology, Drug regulation

## Abstract

Cytochrome P450 (CYP)3A is the most abundant CYP enzyme in the human liver, and a functional impairment of this enzyme leads to unanticipated adverse reactions and therapeutic failures; these reactions result in the early termination of drug development or the withdrawal of drugs from the market. The transcriptional regulation mechanism of the *Cyp3a* gene is not fully understood and requires a thorough investigation. We mapped the transcriptome of the *Cyp3a* gene in a mouse model. The *Cyp3a* gene was induced using the *mPXR* activator pregnenolone-16alpha-carbonitrile (PCN) and was subsequently downregulated using lipopolysaccharide (LPS). Our objective was to identify the transcription factors (TFs), epigenetic modulators and molecular pathways that are enriched or repressed by PCN and LPS based on a gene set enrichment analysis. Our analysis shows that 113 genes were significantly upregulated (by at least 1.5-fold) with PCN treatment, and that 834 genes were significantly downregulated (by at least 1.5-fold) with LPS treatment. Additionally, the targets of the 536 transcription factors were enriched by a combined treatment of PCN and LPS, and among these, 285 were found to have binding sites on *Cyp3a11*. Moreover, the repressed targets of the epigenetic markers HDAC1, HDAC3 and EZH2 were further suppressed by LPS treatment and were enhanced by PCN treatment. By identifying and contrasting the transcriptional regulators that are altered by PCN and LPS, our study provides novel insights into the transcriptional regulation of CYP3A in the liver.

## Introduction

Cytochrome P450 3A (CYP3A) is the most abundant subfamily of the drug-metabolizing enzymes (DMEs) that are responsible for the disposition of more than 50% of the currently prescribed drugs^1–4^. A review of 121 new molecular entities (NMEs), approved by the FDA in 2003 and 2008, indicated that CYP3A was the main CYP enzyme involved in the disposition of these NMEs^5^. The clinical importance of CYP3A can be assessed from numerous reports showing that the downregulation of *CYP3A* expression and activity in infectious and inflammatory diseases and in liver cancer^6,7^ leads to the failure of therapy and/or potentially harmful adverse drug reactions^8,9^. On the other hand, the induction of CYP3A4 is associated with the reduced efficacy of clinically relevant medications^10–12^. Therefore, to reduce or prevent unwanted drug-drug interactions and adverse drug reactions, it is crucial to gain a comprehensive understanding of the molecular mechanisms that regulate the CYP3A enzyme.

CYP3A is both constitutively expressed and transcriptionally induced or inhibited by a variety of structurally diverse xenobiotics. Multiple signaling pathways contribute to the complex regulation of the *CYP3A* genes. The constitutive expression of CYP3A is regulated via basal transcription factors, such as HNF4, HNF1, AP1, C/EBPα, C/EBPβ, HNF3γ, and USF1, by binding to the constitutive liver enhancer module (CLEM4) and the distal enhancer module (XREM) of the *CYP3A4* promoter^13–18^. The xenobiotic-mediated induction of *CYP3A* is indirect and involves the activation of nuclear receptors, such as pregnane X receptor (PXR), constitutive androstane receptor (CAR), glucocorticoid receptor (GR) and vitamin D receptor (VDR)^19,20^. However, PXR is considered the most important and critical determinant of hepatic CYP3A enzyme activity and expression^21,22^. PXR is expressed in the cytosol and is activated upon binding with structurally diverse drug ligands, including barbiturates, rifampicin, statins, pregnenolone 16α-carbonitrile (PCN) and many others. Upon activation, PXR is translocated to the nucleus, where it heterodimerizes with retinoid X receptor (RXR) and enhances *CYP3A* transcription by binding to AGGTCA-like direct repeat (DR-3) and everted repeat regions (ER-6) on the *Cyp3a* gene^22–25^. PXR activity can be modulated by phosphorylation through a number of cell signaling kinases, such as protein kinase A^26,27^, protein kinase C^28^, c-Jun-N-terminal kinase^29^, and this impacts its downstream transcriptional ability to induce *CYP3A*. Epigenetic changes, such as DNA methylation, histone protein modification and microRNAs (miRNAs) have also been implicated in the regulation of the CYP3A enzyme.

In contrast to induction of *CYP3A* being xenobiotic-mediated, the downregulation of hepatic *CYP3A* has mainly been reported in various pathophysiological conditions, especially infections and inflammation. Studies have shown that the gram-negative bacterial endotoxin lipopolysaccharide (LPS) induces an acute phase response^30^ in animals, and this response can lead to the decreased expression and activity of CYP3A11^31,32^; ultimately, this leads to a decrease in the hepatic drug metabolism^33^. Multiple mechanisms have been proposed to explain the effects of LPS on *CYP3A* downregulation. LPS treatment of mice suppresses the *PXR* mRNA levels and reduces the nuclear RXRα protein levels due to increased nuclear export^34^. The binding of PXR/RXRα to conserved sequences of *Cyp3a11* was also reduced by LPS, thereby suppressing CYP3A11 mRNA^34^. LPS has also been shown to activate toll-like receptors (TLRs) on hepatocytes and Kupffer cells, leading to the induction of pro-inflammatory cytokines, such as IL-1β, IL-6 and TNF-α in immune cells^35,36^. In turn, these increased levels of cytokines downregulate *Cyp3a* gene expression by activating downstream mediators, such as JNK or NF-κB^37–39^. The translocation of NF-κB was shown to increase binding between NF-κB and RXRα, and this increase interfered with the formation of PXR-RXRα and suppressed CYP3A4 expression^40^.

Although numerous mechanisms, both *in vitro* and *in vivo*, have been proposed to explain the altered *CYP3A* expression levels, global transcriptome changes have not yet been investigated. We utilized the model of CYP3A upregulation by PCN (mouse-specific PXR activator) followed by CYP3A downregulation by LPS and performed a comprehensive transcriptome mapping and bioinformatics analysis to identify the novel mechanisms of CYP3A11 (mouse homolog of CYP3A4) regulation *in vivo*. Our study identified genes, gene pathways, transcription factors and epigenetic modulators that were significantly altered (induced or downregulated) by PCN and LPS. By comparing and contrasting the effects of PCN and LPS on the transcriptome as a whole, and by finding transcription factors and epigenetic modulators, which are either upregulated or downregulated by PCN or LPS, we identified potential regulators involved in the *Cyp3a* transcriptional machinery, which can be targeted for further investigation.

## Results

### CYP3A11 expression and activity

**Figure 1:**
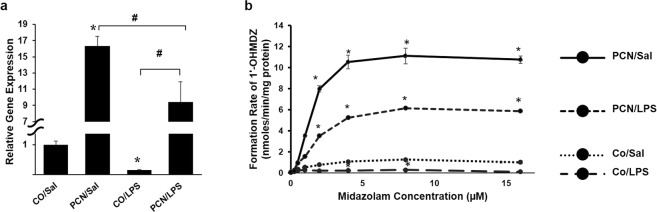
Validation of *Cyp3a11* modulation by PCN and LPS. (**a**) The real-time RT-qPCR analysis of *Cyp3a11* gene expression is shown. (**b**) The CYP3A11 enzyme activity is shown from the livers of mice treated with corn oil or PCN (50 mg/kg/day) for 3 days followed by saline or LPS (2 mg/kg) for 16 h. The error bars represent the standard deviation from three independent experiments performed in quadruplicate. *p < 0.05 compared to the control treatment. #p < 0.05 compared to PCN or LPS treatment alone.

To validate our model, we analyzed the gene expression of *Cyp3a11* in the mouse liver after treatment with PCN and LPS using RT-qPCR. We observed that treatment with PCN upregulated *Cyp3a11* gene expression by 16-fold, whereas LPS treatment downregulated *Cyp3a11* gene expression by 10-fold compared to the control gene expression (Fig. 1a). The combined treatment of PCN and LPS induced a significantly higher expression level of *Cyp3a11 c*ompared to that induced in the control; however, its expression was reduced by almost 1.7-fold by the combination of PCN and LPS compared to the expression by PCN treatment alone. A microarray analysis of the transcriptomic profile also showed that PCN significantly upregulated *Cyp3a11* gene expression 2.41-fold, whereas LPS significantly downregulated *Cyp3a11* gene expression 2.6-fold (Tables 1 and S1); these results are in concordance with the RT-qPCR data. The CYP3A11 activity was measured by the formation rate of the metabolite of midazolam (MDZ) as described in the Materials and Methods section. Consistent with the expression data, CYP3A11 activity was significantly induced by PCN and was downregulated by LPS (Fig. 1b). The combined treatment of PCN and LPS significantly induced Cyp3a11 activity compared to that in the control and attenuated Cyp3a11 activity compared to that in the individual PCN treatment.

**Table 1:** Top 15 differentially regulated genes (represented by their gene symbols) by PCN, LPS and combined PCN/LPS treatments compared to those in the control.

PCN vs Control		LPS vs Control		PCN/LPS vs Control		PCN/LPS vs PCN/Sal		PCN/LPS vs CO/LPS	
Up	Down	Up	Down	Up	Down	Up	Down	Up	Down
GSTA1	CYP4A14	REG3B	CAR3	S100A8	HSD3B5	CXCL9	THRSP	CYP3A11	CYP4A14
GSTM3	CML2	S100A8	HSD3B5	S100A9	HAMP2	SAA3	CAR3	CES6	FDPS
CYP2C55	EGFR	S100A9	CPS1	SAA3	CAR3	S100A9	AQP8	GSTA1	IDI1
CES6	EGR1	SAA3	THRSP	REG3B	AQP8	S100A8	HAMP2	CYP3A25	REG3A
GSTA2	ARRDC3	CXCL9	HAMP2	CRYBB3	THRSP	REG3B	HSD3B5	GSTM3	SC4MOL
CYP2B10	IDB2	CRYBB3	ELOVL3	CXCL9	VSIG4	CRYBB3	ELOVL6	GSTA2	SLC25A25
AKR1B7	CYP2C67	CXCL1	VSIG4	CXCL1	ELOVL3	CD14	GSTA1	POR	CYP51
CYP3A11	GNAT1	CD14	INMT	CD14	CPS1	CXCL1	CYP2C55	HSD17B6	PPP1R3C
GSTM6	G0S2	LCN2	ACSS2	LCN2	ACSS2	CCL5	G6PC	CYP2C55	LSS
GSTM2	RNASE4	ADH7	AQP8	MT2	G6PC	LCN2	ACSS2	AKR1B7	CRELD2
HSD17B6	ACOT1	MT2	CLEC4G	SAA2	CLEC4G	SAA2	GSTM3	CYP2B10	
CYP2B23	AOX3	CPNE8	CYP2A5	CHI3L3	SLC2A2	MT2	SLC2A2	GSTM2	
DDIT4	CYP2C70	MT1	UPP2	CES6	ACAA1B	CYP17A1	CHRNA4	SLCO1A4	
CYP3A25	HSD3B	SAA2	G6PC	ADH7	NUDT7	GBP2	CPS1	GSTM6	
CSAD	MUG2	STEAP4	GSTM6	MT1	AOX3	CHI3L3	GSTM6	CES3	

### Differential gene expression analysis

**Figure 2:**
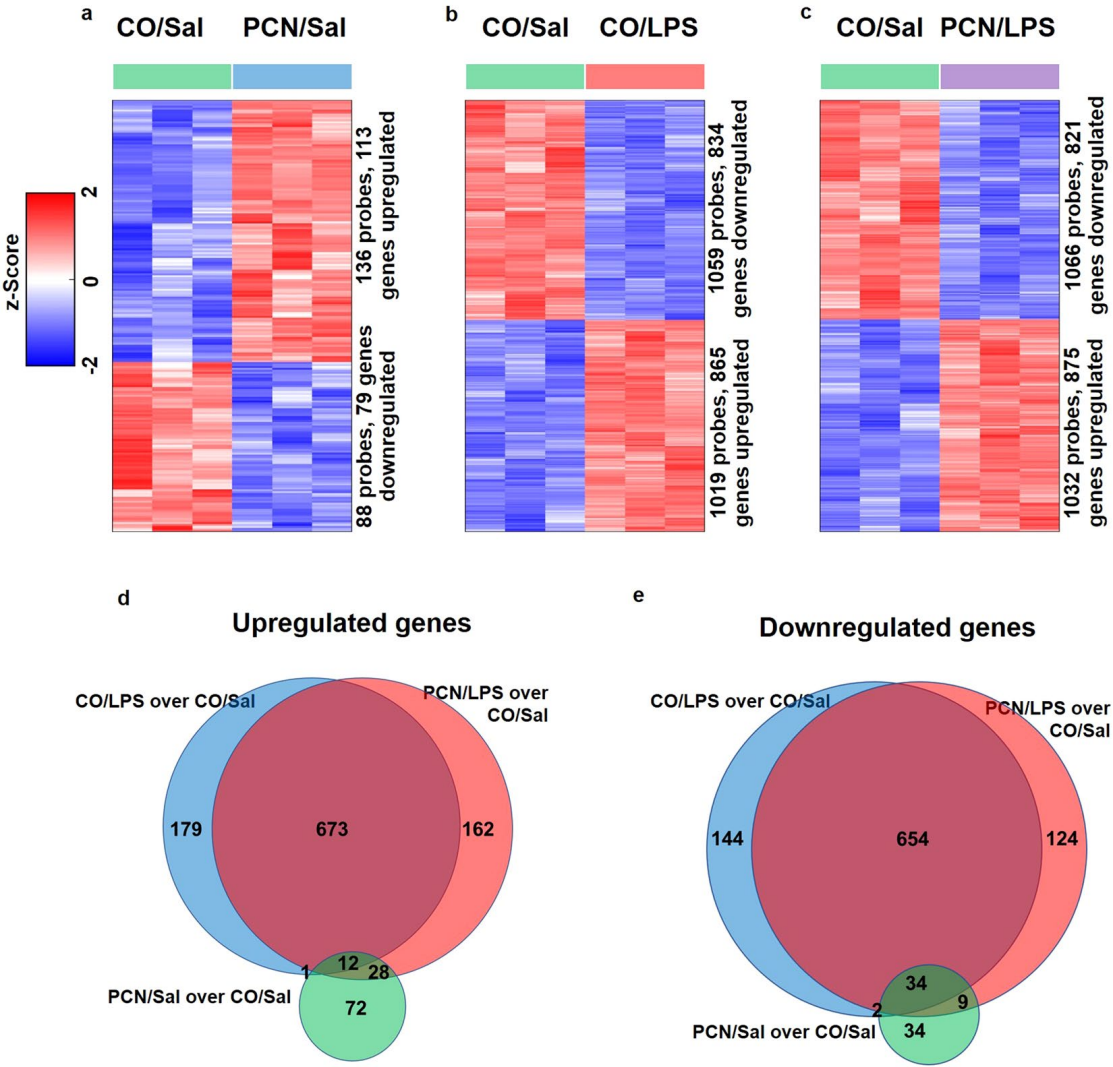
PCN and LPS treatments lead to robust yet distinct transcriptomic changes. Heatmaps of the differentially expressed genes (DEGs) with **(a)** exclusive PCN treatment, **(b**) exclusive LPS treatment and **(c)** combined PCN/LPS treatment are shown. (**d**–**e**) The number of upregulated and downregulated DEGs by PCN treatment, LPS treatment and combined PCN/LPS treatment compared to those in the control are shown. The genes were considered to be differentially expressed with a p-value < 0.05 and a fold change greater than or equal to 1.25-fold or less than or equal to 0.8-fold. Heatmaps were generated using mean-centered normalized expression values (z-scores), employing the Euclidean distance metric, the average clustering method and R statistical software.

A gene expression analysis using a DNA microarray was carried out to identify genes and pathways that are upregulated by PCN and downregulated by LPS or downregulated by PCN and upregulated by LPS. After three days of PCN treatment, a total of 79 genes were downregulated (DR: 79), and 113 genes were upregulated (UR: 113) (Fig. 2a). However, after a 16 h LPS treatment, 834 genes were downregulated, and 865 genes were upregulated (Fig. 2b). With the combined PCN and LPS treatment, a total of 821 genes were downregulated, and 875 genes were upregulated compared to those in the control group (Fig. 2c). Figure 2d,e show the number of upregulated and downregulated genes in the three treatments compared to those in the control. Among these total changes, Table 1 represents the top 15 genes with the highest fold change; these genes were differentially expressed among all the global changes upon PCN and/or LPS treatment compared to those in the control. PCN treatment led to significant alterations in the gene expression of numerous drug metabolizing enzymes, such as glutathione S transferases, CYP3A11, CYP2B10, and carboxylesterases. On the other hand, LPS upregulated many inflammatory mediators, such as chemokines and CD14. Interestingly, with the combined treatment of PCN and LPS, we observed similarities in the top up- and downregulated genes as observed in genes treated with LPS alone.

### Pathway analysis

**Figure 3:**
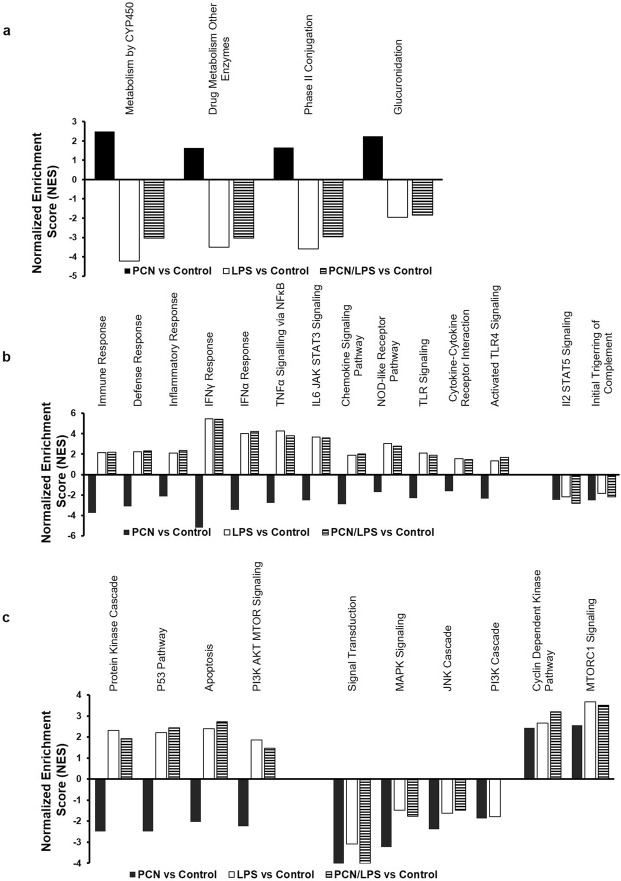
Gene set enrichment analysis (GSEA) reveals a distinct modulation of pathways between the PCN and LPS treatments. The mice were treated with corn oil or PCN (50 mg/kg/day) for 3 days followed by saline or LPS (2 mg/kg) for 16 h. The biological processes enriched in the transcriptome footprint of liver mRNA from the treated mice were identified using gene set enrichment analysis (GSEA). The normalized enrichment score (NES) is reported for select enriched pathways (FDR-adjusted Q-value < 0.25). The key differences were observed in (**a**) drug metabolism pathways, (**b**) inflammatory pathways and (**c**) signal transduction pathways.

Biological processes that were significantly enriched but differentially modulated in the transcriptome footprint of the two treatments, PCN and LPS, were identified using gene set enrichment analysis (GSEA)^41^. Among the overall enriched pathways, we identified those that were regulated in opposite directions by each of the PCN and LPS treatments (Q < 0.25; normalized enrichment score/NES has opposite signs between the PCN and LPS treatments). In addition, we studied the effect of the cotreatment of PCN and LPS on these differentially regulated pathways, as shown in Fig. 3. We focused on three groups of major biological pathways involved in drug response biology: (a) drug metabolism pathways, including P450-dependent metabolism or glucuronidation (Fig. 3a); (b) inflammatory pathways, including interferon-γ, interferon-α, and TNF-α signaling (Fig. 3b); and (c) signal transduction pathways, including the protein kinase cascade and mitogen-activated protein kinase signaling (Fig. 3c). The drug metabolism pathways were positively enriched by PCN and were attenuated by LPS, whereas the LPS/PCN combination suppressed the effects of the single PCN treatment. On the other hand, both the inflammatory pathways and the signal transduction pathways were mainly negatively enriched by PCN and were positively enriched by LPS. Similar to the pattern observed in DEGs with the combined PCN and LPS treatment, most of the pathways were enriched in the same direction as the LPS treatment alone. Only a handful of inflammatory pathways were modulated in the same direction by both PCN and LPS, such as IL2 and STAT5 signaling. However, among the signal transduction processes, most of the pathways, including MAPK signaling, the JNK cascade, and the PI3K cascade, were similarly suppressed by both PCN and LPS treatments, whereas cyclin-dependent kinase and MTORC1 signaling were induced by both PCN and LPS treatments.

### Transcription factor analysis

**Figure 4:**
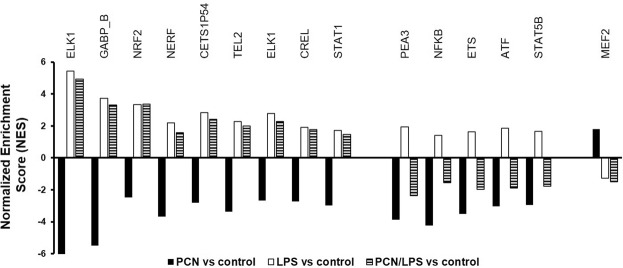
Gene set enrichment analysis (GSEA) reveals the distinct modulation of transcriptional regulators. The enrichment of transcriptional regulators in the transcriptomic response of mouse livers exposed to corn oil or PCN (50 mg/kg/day) for 3 days followed by saline or LPS (2 mg/kg) for 16 h was assessed using GSEA. An extensive search was carried out for transcriptional regulators that were enriched (FDR-adjusted Q-value < 0.25) but with targets changed in the opposite direction between the PCN and LPS treatments compared to those in the control. We report transcriptional regulators with a positive NES (acting primarily as transcriptional activators for *Cyp3a11*) and those with a negative NES (acting primarily as transcriptional repressors for *Cyp3a11*).

Next, we analyzed the transcription factors (TFs) that may have a role in mediating the changes in the expression of hepatic genes in mice treated with PCN +/− LPS. Using GSEA, we identified transcription factors whose targets were differentially enriched by the individual PCN or LPS treatment (Fig. 4). The top transcription factors that may be involved in the expression of upregulated and downregulated genes are shown in Table 2. After three days of PCN treatment alone, a total of 563 transcription factors were negatively enriched, and only 3 transcription factors were positively enriched, i.e., myocyte enhancer factor 2 (MEF2), nuclear factor erythroid 2 (NFE2) and peroxisome proliferator-activated receptors γ (PPARγ). Among these three, MEF2 was the only transcription factor that was also negatively enriched with LPS. However, after 16 h of LPS treatment, 472 transcription factors were negatively enriched, and 65 transcription factors were positively enriched. In the PCN/LPS group, 536 TFs were differentially expressed (upregulated: 35, downregulated: 501) compared to those in CO/Sal. Using a TRANSFAC-based motif analysis, we identified TFs that are altered by PCN or LPS (in the same or opposite direction) and might bind to *Cyp3a11* (Table 3), and to the *CYP3A4* promoter sequence (Supplementary Table S2). We found that among the 536 TFs, 285 had potential binding sites on *Cyp3a11*, including Stat1, Stat5b, Pax4, Mycmax and Pea3, along with some known mediators, such as HNF1, HNF3 and CREB.

**Table 2:** Top 15 differentially enriched transcription factors by PCN, LPS and combined PCN/LPS treatments compared those in the control.

PCN vs Control		LPS vs Control		PCN/LPS vs Control		PCN/LPS vs PCN/Sal		PCN/LPS vs CO/LPS	
Up	Down	Up	Down	Up	Down	Up	Down	Up	Down
MEF2	SP1	ELK1	FOXO4	ELK1	FOXO4	ELK1	FOXO4		SP1
PPARG	ELK1	GABP_B	SP1	NRF2	FREAC2	GABP_B	SP1		MAZ
NFE2	FOXO4	NRF2	FREAC2	GABP_B	SP1	NRF2	FREAC2		E12
	MAZ	CETS1P54	E12	CETS1P54	NFAT	ETS2_B	MAZ		LEF1
	ETS2_B	TEL2	NFAT	TEL2	MAZ	STAT5B	LEF1		NFY
	LEF1	NFKB	MYC	NFKAPPAB65	LEF1	HNF4	AP4		FOXO4
	E12	COUP	MAZ	NFE2	E12	CETS1P54	NFAT		NFAT
	GABP_B	AP1	AP4	CREL	AP4	NERF	MEF2		ELK1
	NFAT	PEA3	LEF1	IRF	MYOD	TEL2	E12		MYOD
	FREAC2	CREL	HNF3	MAX	ERR1	IRF	MYOD		LEF1
	AP4	ATF	ERR1	BACH1	LEF1	STAT5A	MYC		MEIS1
	HNF3	CREB	HNF1	USF	MYC	NFKB	ERR1		AP4
	PU1	BACH1	CHX10	ICSBP	CHX10	PU1	CHX10		FREAC2
	PAX4	MYCMAX	MYOD	AP1	HNF3	ICSBP	NFY		PU1
	MYC	ATF6	NKX25	NERF	MEF2	SP1	MEIS1		E4F1

**Table 3:** Altered transcription factors predicted to have putative binding sites on the Cyp3a11 promoter using TRANSFAC analysis.

PCN↓ LPS↓	PCN↓ LPS↑
FREAC2	NFKB
NFAT	PEA3
LEF1	STAT1
**HNF**1	STAT5B
CHX10	AP2
ERR1	CETS1P54
**HNF3**	**CREB**
MYOD	PAX4
GATA1	**COUP**
FOXO4	HSF
USF2	MYCMAX
PAX4	PTF1BETA
NFY	
NF1	
SREBP1	

### Analysis of epigenetic changes

**Figure 5:**
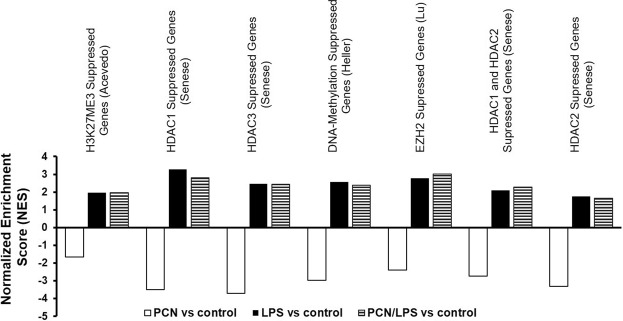
Gene set enrichment analysis (GSEA) reveals distinct patterns of change for epigenetic modulators. The enrichment of epigenetic mechanisms in the mouse livers exposed to corn oil or PCN (50 mg/kg/day) for 3 days followed by saline or LPS (2 mg/kg) for 16 h was assessed using GSEA. An extensive search was carried out for epigenetic regulators that were enriched (FDR-adjusted Q-value < 0.25) but with targets changed in the opposite direction between the PCN and LPS treatments compared to those in the control.

To understand the role of epigenetic modulators in regulating *Cyp3a11* at the transcriptional level, a GSEA was carried out against the Molecular Signature Database (MSigDB) compendium of annotated gene sets^42^. Figure 5 shows the targets of epigenetic modulators that are significantly enriched but in opposite directions by a single treatment with either PCN or LPS. The targets of methylation by modulators, such as HDAC1, HDAC3, EZH2, H3K27ME3, were suppressed by PCN, but the suppression was reversed by the combined LPS and PCN treatment. Changes by these epigenetic modulators have been reported in numerous *in vitro* and *in vivo* models, and we believe that the same epigenetic modulators could also be involved in the regulation of *Cyp3a11* expression and activity in models treated with PCN and LPS.

### Real-time qPCR

**Figure 6:**
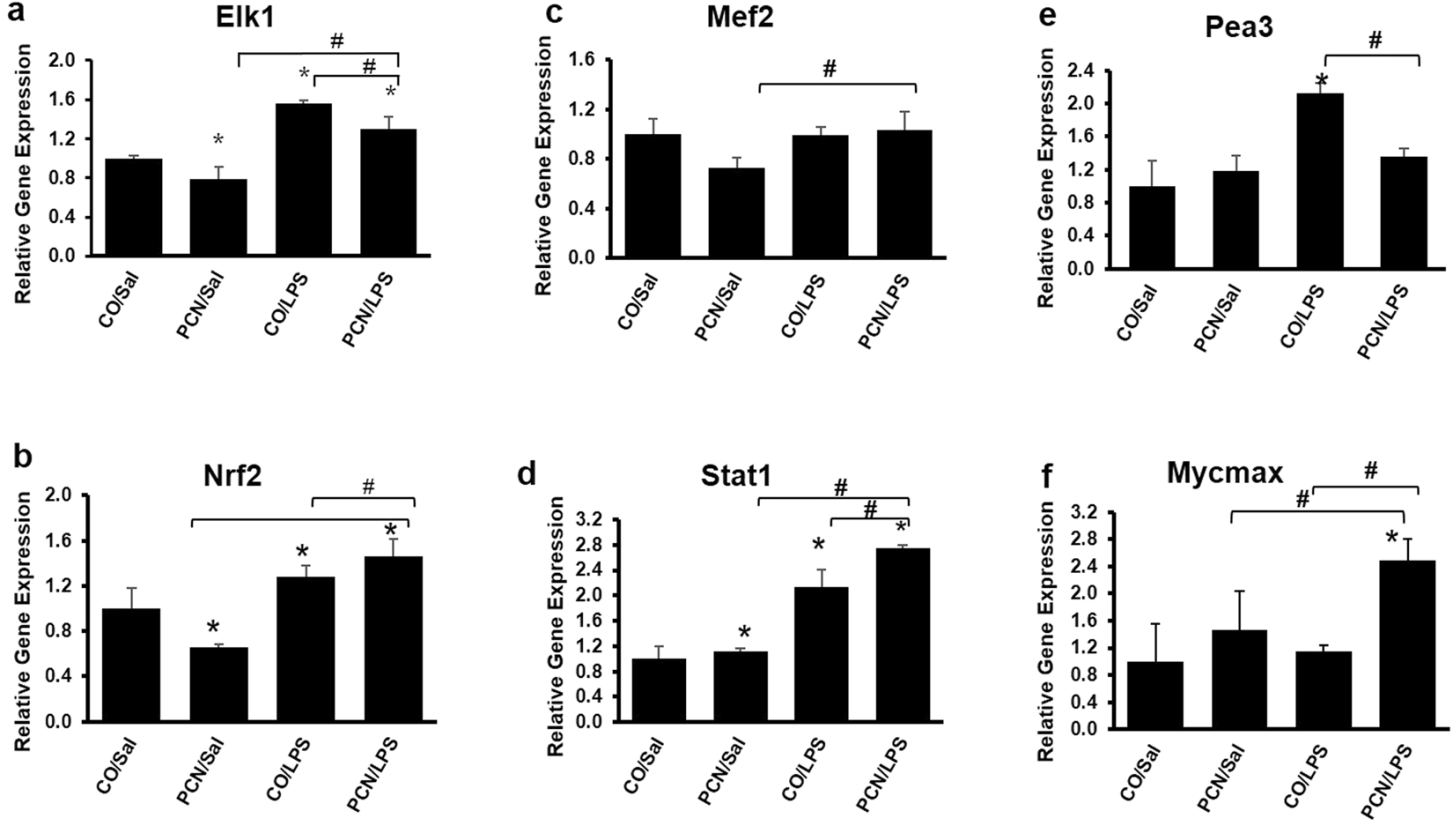
Real-time qPCR analysis for the validation of gene expression of the following transcription factors: (**a**) Elk1, (**b**) Nrf2, (**c**) Mef2 (**d**) Stat1, (**e**) Pea3, and (**f**) Mycmax. The mice were treated with corn oil or PCN (50 mg/kg/day) for 3 days followed by saline or LPS (2 mg/kg) for 16 h. A few select candidate genes from the list of transcription factors that were differentially regulated between the PCN and LPS treatments were chosen for the validation of gene expression. *p < 0.05 compared to the control treatment. ^#^p < 0.05 compared to PCN or LPS treatment alone.

The microarray results were validated by RT-qPCR. Instead of choosing targets based solely on fold change for validation, we selected candidate genes from the list of transcription factors and epigenetic modulators that were differentially regulated between the PCN and LPS treatment groups. Two transcription factors, Elk1 and Nrf2, were selected for validation because they were maximally enriched in opposite directions by PCN and LPS. As shown in Fig. 6a,b, there was good concordance between the microarray and the RT-qPCR data for both Elk1 and Nrf2, as both are significantly downregulated by PCN and are upregulated by LPS. We also validated the expression levels of Mef2, Stat1, Pea3 and Mycmax because these transcription factors were differentially enriched by PCN and LPS in opposite directions and also might bind to the *Cyp3a11* promoter according to TRANSFAC analysis. The expression of both Stat1 and Pea3 was induced by LPS treatment, and there was good concordance between the microarray analysis and RT-qPCR data, suggesting that Stat1 and Pea3 might be negative regulators of *Cyp3a*.

**Figure 7:**
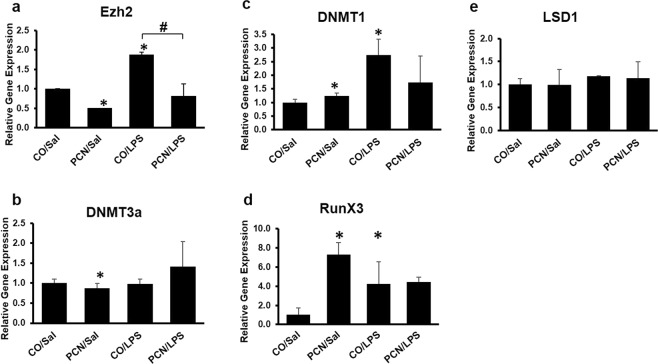
Real-time qPCR analysis for the validation of gene expression of the following epigenetic modulators: (**a**) Ezh2, (**b**) DNMT3a, (**c**) DNMT1, (**d**) RunX3 and (**e**) LSD1. The mice were treated with corn oil or PCN (50 mg/kg/day) for 3 days followed by saline or LPS (2 mg/kg) for 16 h. A few select candidate genes from the list of epigenetic factors that were differentially regulated between the PCN and LPS treatments were chosen for the validation of gene expression. *p < 0.05 compared to the control treatment. ^#^p < 0.05 compared to PCN or LPS treatment alone.

We also carried out an RT-qPCR analysis to investigate whether the actual gene expression of epigenetic markers is altered by PCN and/or LPS. We found that both EZH2 and DNMT3a were significantly downregulated with PCN treatment compared to those in the control; this is in accordance with our GSEA data (Fig. 7a,b). The combined treatment of PCN and LPS significantly reduced the gene expression of EZH2, thereby showing that PCN can attenuate the effect of LPS by regulating targets of EZH2. However, the gene expression of DNMT1 and RunX3 was significantly induced by PCN and LPS (Fig. 7c,d).

## Discussion

In this study, we identified novel signaling pathways, transcription factors and epigenetic mechanisms that are potentially involved in the regulation of *Cyp3a11* (mouse homolog of *CYP3A4*). Numerous mechanisms that alter the drug metabolizing enzymes (DMEs), especially *CYP3A11*, have been reported, but a comprehensive study of all the transcriptomic changes associated with the upregulation and downregulation of CYP3A11 has not been carried out. We found robust changes in the mouse genomic profile upon treatment with PCN, the *Cyp3a11* inducer, and LPS, the endotoxin responsible for the downregulation of *Cyp3a11*. PCN treatment leads to the increased binding of PXR to the *Cyp3a* promoter/enhance region leading to *Cyp3a* induction, while LPS attenuates this induction; this is probably due to the reduced binding of PXR and/or additional transcription factors and co-regulators. Therefore, our analysis has led to the identification of genes encoding signaling pathways, transcription factors, coregulators, and epigenetic factors, which regulate changes in *Cyp3a* gene expression. Common genes that are changed by PCN and LPS in opposite directions will likely be involved in modulating Cyp3a upregulation and downregulation. These results will provide the foundation for further studies to identify changes in the binding of these factors to the *Cyp3a* gene and to understand how signaling pathways modulate these factors to change their expression and binding in relation to *Cyp3a* expression.

Genes that had the largest upregulation due to PCN treatment mainly included DMEs, such as *Cyp2c55*, carboxylesterases, *Cyp2b10*, glutathione S-transferases, and aldo-keto reductases. These results are consistent with previous studies that used chromatin immunoprecipitation sequencing (ChIP-Seq) and reported PXR binding sites on glutathione S-transferases^43^, carboxylesterases and most other DMEs. Most studies that showed the effects of PCN on the liver have traditionally focused on the function and inducibility of enzymes involved in drug metabolism. Interestingly, in our transcriptome analysis, PXR simultaneously induced and repressed hundreds of genes apart from DMEs, including epidermal growth factor receptor (EGFR), early growth response protein 1 (EGR1), arrestin domain containing protein 3 (ARRDC3), and cysteine sulfinic acid decarboxylase (CSAD). We therefore believe that further analysis is warranted to confirm our gene expression analysis of the regulators listed in Table 1, as the drugs that bind directly or indirectly (by the activation of PXR) to these novel regulators could further alter the expression of the *Cyp3a11* gene. Since multiple EGR1 binding sites have previously been identified within the 5′-regulatory promoter region of the *CYP2B6* gene^44,45^, its altered gene expression may imply potential involvement in the regulation of *Cyp3a11*. With LPS treatment alone, the gene expression pattern of numerous inflammatory mediators was differentially regulated, including serum amyloid A3 (SAA3), chemokine ligand 9 (CXCL9), cluster of differentiation 14 (CD14), and metallothionein 2 (MT2). The accurate and comprehensive knowledge of regulators that are differentially regulated by LPS can help identify factors that may potentially affect *Cyp3a11*.

Although transcriptome profiling using a DNA microarray has become a mainstay of genomics research^46,47^, the challenge no longer lies in obtaining differential gene expression patterns, but rather, is in interpreting the results to gain insights into the biological mechanisms. A powerful analytical tool is gene set enrichment analysis (GSEA), in which all expressed genes are ranked according to their differential expression, then the enrichment scores for each pathway or gene set of interest are computed. The normalized enrichment scores (NES) and the statistical measures of significance (P-value and FDR-adjusted Q-value) for specific pathways or gene sets are determined by performing 1000 permutations of the rank file, re-computing the enrichment score for each gene set and permutation, and finally integrating the results of all 1000 permutations. GSEA then determines the degree of representation of the members in a gene set by ranking them in a list from the top (positive enrichment) to the bottom (negative enrichment)^41^. The transcriptome profiling of mouse livers after PCN and LPS treatments robustly identified numerous genes, including some genes with functions related to drug metabolism, cell cycle kinetics and inflammation mediation. We broadly selected pathways that belong to the following three major mechanisms for enrichment analysis: drug metabolism (DM), inflammatory regulation (IR) or signal transduction (ST). We observed that most of the drug metabolism pathways were positively enriched by PCN and were downregulated by LPS; this is consistent with the changes in *Cyp3a11* gene expression. Taking a closer look into the subsets of genes belonging to these pathways, we found that although *Cyp3a11* is positively enriched, multiple drug metabolizing enzymes, such as glutathione S transferase A3, aldo-keto reductase 1C6 or alcohol dehydrogenase 1 are negatively enriched; this negatively shifts the total enrichment score of the pathway by treatment with either PCN or LPS. In contrast to the DM pathways, most of the IR and ST pathways were found to be negatively enriched by PXR activation and positively enriched by LPS treatment and by the combined treatment. Understanding which pathways are enriched by PCN and LPS is crucial because this may affect the components involved in the transcriptional regulation of not only *Cyp3a11* but also other DMEs.

The gene transcription of *Cyp3a11* is largely regulated by transcription factor (TF) proteins that bind to genomic cis-regulatory elements that are characterized by precise DNA motifs. Changes in the gene expression of TFs are usually not detected in microarray experiments, because their activity is primarily regulated by ligand binding or by posttranscriptional modifications^48^. We employed GSEA to find the top transcription factors whose targets were enriched in opposite directions by PCN and LPS. One of the top transcription factors was Elk1, an ETS family transcription factor that is responsible for target gene transcription upon mitogen-activated protein kinase-signaling pathway stimulation^49^. Elk1 was downregulated by PCN and was upregulated by LPS, and the combined treatment followed the LPS response. In fact, most of the transcription factors, such as Tel2, Pea3, Stat1, and Stat5b that were changed in opposite directions were suppressed by PCN and were positively enriched by LPS treatment; this suggests that these transcription factors might negatively regulate basal *Cyp3a11* expression. This fact was strengthened by previous reports showing that the loss of Stat5b increased the gene expression of *Cyp3a* in mice^50^. LPS-mediated activation of NF-κB has also been shown to play a significant role in the downregulation of the Cyp3a enzyme^40,51^. On the other hand, Mef2 was the only transcription factor that was positively enriched by PCN and was attenuated by LPS in our study. Mef2 regulates cell differentiation, proliferation, morphogenesis, survival and apoptosis in a wide range of cell types^52^, and a previous microarray analysis has revealed that a few DMEs, such as CYP1B1, and nuclear receptors, such as Ahr, are downregulated in the absence of Mef2^53^. Although the actual gene expression of *Mef2* was not induced by PCN in our data, it might still be involved in altering the expression of its downstream genes. Further studies to understand the role of Mef2 in the regulation of the CYP3A enzyme need to be carried out.

Furthermore, a TRANSFAC analysis was carried out to investigate whether these enriched transcription factors have any binding sites on the *Cyp3a* promoter and enhancer regions. TRANSFAC (TRANScription FACtor database) is a manually curated database of eukaryotic transcription factors, their genomic binding sites and their DNA binding profiles. The contents of this database can be used to predict potential transcription factor binding sites. Some transcription factors that are already known to bind to *Cyp3a*, e.g., HNF1, HNF3, CREB and COUP, were also identified by TRANSFAC, validating our analysis. Other transcription factors that may have potential binding sites on Cyp3a are listed in Table 3, and RT-qPCR was performed to investigate whether PCN and LPS alter the gene expression of these transcription factors. The data from this analysis provide novel insights into the mechanisms involved in the regulation of human *CYP3A4* and suggest new therapeutic targets to treat disorders that are caused by an altered drug metabolism.

Lastly, recent studies have demonstrated that many other factors, such as epigenetics^54^ and micro RNAs (miRNAs)^55^, may modulate DME gene expression and may cause variations in drug metabolism and toxicity. The effect of epigenetic processes on pharmacologically relevant genes and, ultimately, drug response is a relatively new area of research^56^. Among all the epigenetic changes, changes in the DNA methylation profiles determine whether there is a permissive chromatin state for the transcription machinery to access the gene promoter regions and to initiate transcription^57,58^. DNA methylation is a key epigenetic mechanism and a covalent modification that results in stable gene silencing^59^. In our data, we found that genes that are suppressed by DNA methylation by modulators, such as enhancer of zeste homolog 2 (Ezh2) in previous studies^60^, were further suppressed by PCN, and this effect was relieved by LPS. We further carried out an RT-qPCR analysis to measure the gene expression of the methylation modulator Ezh2 in our model. We found that Ezh2 gene expression was suppressed by PCN and was induced by LPS; these results indicate that Ezh2 could also have a significant role in the regulation of the CYP3A enzyme. Future studies to determine whether Ezh2 methylates *Cyp3a11* need to be conducted. Enrichment of histone-3-lysine-27 trimethylation (H3K27me3) in promoters and gene bodies has also been associated with the inactivation of gene transcription^61,62^. Li *et al*. found that increased H3K27me3 within the margins of the *Cyp3a16* gene may be responsible for switching off *Cyp3a16* gene expression in the livers of adult mice^63^. In addition to being homologous to the human CYP3A isoforms in DNA and protein sequences, the mouse *Cyp3a11* and *Cyp3a16* homologues also mimic a developmental switch, such as human *CYP3A4* and *CYP3A7* ^64^. In our GSEA, we found that the genes that were downregulated in liver tumors by H3K27me3^65^ were further suppressed by PCN and relieved by LPS. This could imply that although H3K27me3 might be responsible for the switch of *Cyp3a16* to *Cyp3a11*, high levels of H3K27me3 could be responsible for the decreased expression of *Cyp3a11* in the adult liver. However, further methylation-specific studies need to be carried out to confirm the involvement of H3K27me3 in the regulation of *Cyp3a11* in adult mice. Apart from epigenetic modulation, microRNA-27b (miR-27b) and mouse microRNA-298 (mmu-miR-298) have previously been shown to downregulate *CYP3A4* expression^66^. Hence, GSEA was performed to understand the involvement of miRNAs in the regulation of *Cyp3a11*; however, PCN and LPS did not significantly enrich any miRNAs in opposite directions in our model (data not shown). It is possible that LPS can change the miRNA expression at different time-points, and LPS is known to have a temporal effect on the expression of miRNAs and genes.

In conclusion, we carried out a whole-transcriptome analysis to understand the novel molecular mechanisms that are associated with the downregulation of the *Cyp3a11* enzyme. Using high-throughput microarray technology, we screened a large number of genes to detect changes stimulated by individual PCN or LPS treatments, as well as by their combined treatment. Potential transcription factors that are altered by PCN and LPS in opposite directions and might be involved in the regulation of the *Cyp3a* gene were identified, such as Pea3 and Stat1. Their differential expression was validated, and future studies will entail chromatin immunoprecipitation assays to investigate their binding to the *Cyp3a* promoter. The results from this study further enhance our understanding of the intricate network of different cell signaling pathways and epigenetic mechanisms with nuclear receptors, such as PXR. In addition, *Cyp3a* might be a potential target of DNA methylation by epigenetic modulators, such as Ezh2; hence, its exact role needs to be further investigated. Since PXR is involved in the regulation of several DMEs other than CYP3A, these novel pathways, transcription factors and epigenetic modulators could be involved in the regulation of numerous other genes controlled by PXR.

## Materials and Methods

### Reagents and materials

5-Pregnen-3β-ol-20-one-16α-carbonitrile (#P0543) was purchased from Sigma-Aldrich (St. Louis, MO). Lipopolysaccharide (*E*. *coli*, #tlrl-pslta) was purchased from InvivoGen (San Diego, CA). The RNeasy Mini Kit (#74104) was obtained from Qiagen (Valencia, CA). A 96-well PCR plate, Roche PCR Master Mix (Roche Diagnostics), TaqMan® primer and probes for Cyp3a11 (FP: GGATGAGATCGATGAGGCTCTG, RP: CAGGTATTCCATCTCCATCACAGT) and cyclophilin (FP: GGCCGATGACGAGCCC, RP: TGTCTTTGGAACTTTGTCTGCA) were purchased from Sigma-Genosys (Houston, TX). A Mouse WG-6 v2.0 expression BeadChip Kit was obtained from Illumina (San Diego, CA). TaqMan® Gene Expression assays with primers and probes for mouse Elk1 (#Mm00468233_g1), Mef2 (#Mm01340842_m1), Nrf2 (#Mm00477784_m1), Pea3 (#Mm00476696_m1), Stat1 (#Mm01257286_m1), and Mycmax (#Mm00487804_m1) were obtained from Thermo Fisher Scientific (#4331182, Waltham, MA). The primers for the epigenetic markers Ezh2, DNMT1, DNMT3a, LSD1 and RunX3 were a kind gift from Dr. Moorthy from the Baylor College of Medicine, Houston, TX.

### Animals and treatments

Adult C57BL/6 mice (~6 weeks, male, Jackson Labs, Stock no. 000664) were allowed to acclimate to the animal care facility for 7 days. The mice were maintained in a temperature- and humidity-controlled environment, and all animal protocols were approved by the Institutional Animal Care and Use Committee (IACUC) at the University of Houston, Houston, TX. All experiments were performed in accordance with the relevant guidelines and regulations of the committee. The mice were fed a standard diet. Both the food and water could be accessed *ad libitum*, and the mice were maintained in a 12 h day/night cycle. The mice were treated with PCN (50 mg/kg/day) or corn oil I.P. for 3 days followed by LPS (2 mg/kg/day) or saline I.P. for 16 h. After treatment, the animals were anesthetized with isoflurane and euthanized by cervical dislocation under deep anesthesia. The liver tissues were harvested for further analysis.

### RNA Isolation

We used a total of 4 animals per treatment group. The total RNA from the liver samples of mice treated with PCN/LPS was isolated using the RNeasy kit according to the manufacturer’s standard protocols (Qiagen, Valencia, CA). Following the total RNA isolation, the sample concentration was assayed using a NanoDrop-8000 (Thermo Scientific, Wilmington, DE, USA), and quality checks were performed using the NanoDrop spectrophotometer and the Agilent Bioanalyzer. RNA quality parameters were as follows: the 260/280 and 260/230 ratios needed to be greater than 1.8. Further, the RNA Integrity Number (RIN) was analyzed using the Agilent Bioanalyzer. The samples needed to have RIN values of 7–10 and with a range of 1–1.5.

### Real-time qPCR analysis

The cDNA was synthesized from the isolated total mRNA using the High Capacity Reverse Transcription Kit from Applied Biosystems. Real-time PCR was performed using an ABI PRISM 7300 Sequence Detection System instrument and software (Applied Biosystems; Foster City, CA) as described previously^34,39,67^. In short, each reaction mixture (total volume of 25 ml) contained 50–100 ng cDNA, 300 nM forward primer, 300 nM reverse primer, 200 nM fluorogenic probe, and 15 ml TaqMan Universal PCR Master Mix. We extrapolated the quantitative expression values from the standard curves, and these values were normalized to the value of cyclophilin.

### Immunoblotting

Whole liver extracts were prepared as described previously^68^, and the protein concentration was determined using the bicinchoninic acid assay according to the manufacturer’s protocol (Pierce, Rockford, IL). Equal amounts of protein (10 mg) were analyzed by SDS-polyacrylamide gel electrophoresis and were transferred onto a nitrocellulose membrane. The membranes were then probed with a rabbit anti-CYP3A11 antibody, followed by probing with a goat anti-rabbit IgG-alkaline phosphatase secondary antibody. The membranes were then washed and incubated with Tropix CDP star nitroblock II ECL reagent as per the manufacturers’ instructions (Applied Biosystems). The membranes were analyzed using a FlourChem FC imaging system (Alpha Innotech). The images were quantified by densitometry using AlphaEase software.

### CYP3A11 enzyme activity assay

The mouse liver microsomes were prepared as described previously^68^, and the protein concentration of the microsomal fractions was determined using a BCA protein assay kit (Pierce, Rockford, IL, USA) using bovine serum albumin (BSA) as the standard. The CYP3A11 enzyme activity was determined in the mouse liver microsomes using the CYP3A substrate midazolam (MDZ) as described previously with minor modifications^69^. The formation of 1′-OHMDZ from MDZ was used as a specific indicator for mouse CYP3A11 activity. In brief, 0.1 mg of total microsomal protein was incubated with MDZ (0–16 μM), 1.3 mM NADPH and reaction cofactors in 50 mM potassium phosphate buffer (pH 7.4). The reaction was initiated by the addition of glucose-6-phosphate dehydrogenase (1-unit mL^−1^). After 5 min, the reactions were stopped by the addition of an equal volume of acetonitrile containing phenacetin as the internal standard (IS). The incubation mixture was centrifuged at 13,000 rpm at 4 °C for 10 min, and the supernatant was transferred to a 96-well autosampling plate for LC-MS/MS analysis. The identity of 1′OHMDZ and IS was verified by comparing with authenticated standards. The data were fit to the standard Michaelis-Menten rate equation.

### Microarray analysis

A total of 250 ng of total RNA was reverse transcribed, and microarray hybridization was performed using the Illumina Gene Expression Mouse WG-6 v2.0 Expression BeadChip Kit at the Laboratory for Translational Genomics at Baylor College of Medicine. The transcriptome profile data were quartile-normalized by the Bioconductor Lumi package. The Lumi package implemented in the R statistical software, version 2.14.1, was used to perform quality control of the signal intensity data on the transcript probes, background adjustment, variance stabilization transformation, and rank invariant normalization. A detection p-value cutoff of 0.01 was required for the normalized intensities to consider a transcript as detected. The differentially expressed genes (DEGs) were selected following the t-test comparisons among the groups of interest using the R statistical system. The genes were considered to be differentially expressed for a p-value < 0.05 and a fold change greater than or equal to 1.25-fold or less than or equal to 0.8-fold. A graphical representation of the DEGs was generated in the form of heatmaps of mean-centered normalized expression values (z-scores) with the Euclidean distance metric and the average clustering method using R statistical software.

### Pathway enrichment and transcription factor analysis

A rank file for each comparison was created based on the log2-fold change of each gene between the respective comparison groups. We next employed the gene set enrichment analysis (GSEA) methodology and software^41^ against the Molecular Signature database (MSigDB) compendium^42^ of gene sets. Gene set enrichment analysis first finds an aggregate gene set score (termed the enrichment score (ES)) and then runs 1000 permutations to establish a background distribution for the ES. The ratio between the ES and the average ES is termed the normalized enrichment score (NES). GSEA determines whether a key component of a pathway or biological process gene set is significantly and preferentially enriched in the upregulated genes (NES > 0, FDR-adjusted Q-value < 0.25) or in the downregulated genes (NES < 0, FDR-adjusted Q-value < 0.25). An established paradigm to generate a hypothesis is that if the NES values for a pathway that compares two different treatments are significant but have opposite signs, then the treatments might direct the pathways in opposite directions. The following pathways were used to determine the enriched pathways: KEGG, Reactome, Hallmark, and GOBP (Gene Ontology Biological Processes). We also used a compendium of putative transcription factors to identify enriched transcription factor targets in the transcriptome footprints analyzed. A TransFac analysis was employed to identify the list of transcription factors that might bind to Cyp3a11 or the CYP3A4 promoter regions.

### Statistical analysis

The real-time PCR data are shown as the mean and are analyzed with Student’s t-test or one-way analysis of variance for all groups, followed by Tukey’s post-hoc test for pairwise comparisons. The significant values are represented as P < 0.05.

## Supplementary information

Supplementary Figures

## Data Availability

The microarray dataset generated during the current study will be made available in the Gene Expression Omnibus (GEO).
