# Supplementary material for: Transcriptomic profiling identifies novel mechanisms of transcriptional regulation of the cytochrome P450 (Cyp)3a11 gene
Source: Sci Rep. 2019 Apr 30;9:6663. doi: 10.1038/s41598-019-43248-w (PMC6491424; doi:10.1038/s41598-019-43248-w)
Supplement: Supplementary file 1 — Supplementary Figures [file 41598_2019_43248_MOESM1_ESM.pdf]

Transcriptomic profiling identifies novel mechanisms of transcriptional regulation of the cytochrome P450 (*Cyp*)*3a11* gene

Guncha Taneja[1]; Suman Maity[2]; Weiwu Jiang[3]; Bhagavatula Moorthy[3]; Cristian Coarfa[4;] Romi Ghose[1]

[1]Department of Pharmacological and Pharmaceutical Sciences, University of Houston, 4849 Calhoun Rd., Houston, TX 77204, USA

[2]Advanced Technology Cores, Baylor College of Medicine, One Baylor Plaza, Houston, TX 77030, USA

[3]Department of Pediatrics, Section of Neonatology, Texas Children's Hospital, Baylor College of Medicine, 1102 Bates Avenue, Suite 530, Houston, TX 77030, USA

[4]Dan L Duncan Comprehensive Cancer Center, Center for Precision Environmental Health, Molecular and Cellular Biology Department, Baylor College of Medicine, One Baylor Plaza, Houston, TX 77030, USA

Corresponding authors: Romi Ghose, Ph.D., email rghose@central.uh.edu

Cristian Coarfa, PhD, email coarfa@bcm.edu

Bhagavatula Moorthy, PhD., email: bmoorthy@bcm.edu

Current affiliation for G.T- DILIsym Services, A Simulations Plus Company, Research Triangle Park, North Carolina, 27709, USA

Supplementary Figures

Supplementary Figure S1. Volcano plot showing the differentially regulated genes by PCN and LPS treatment.

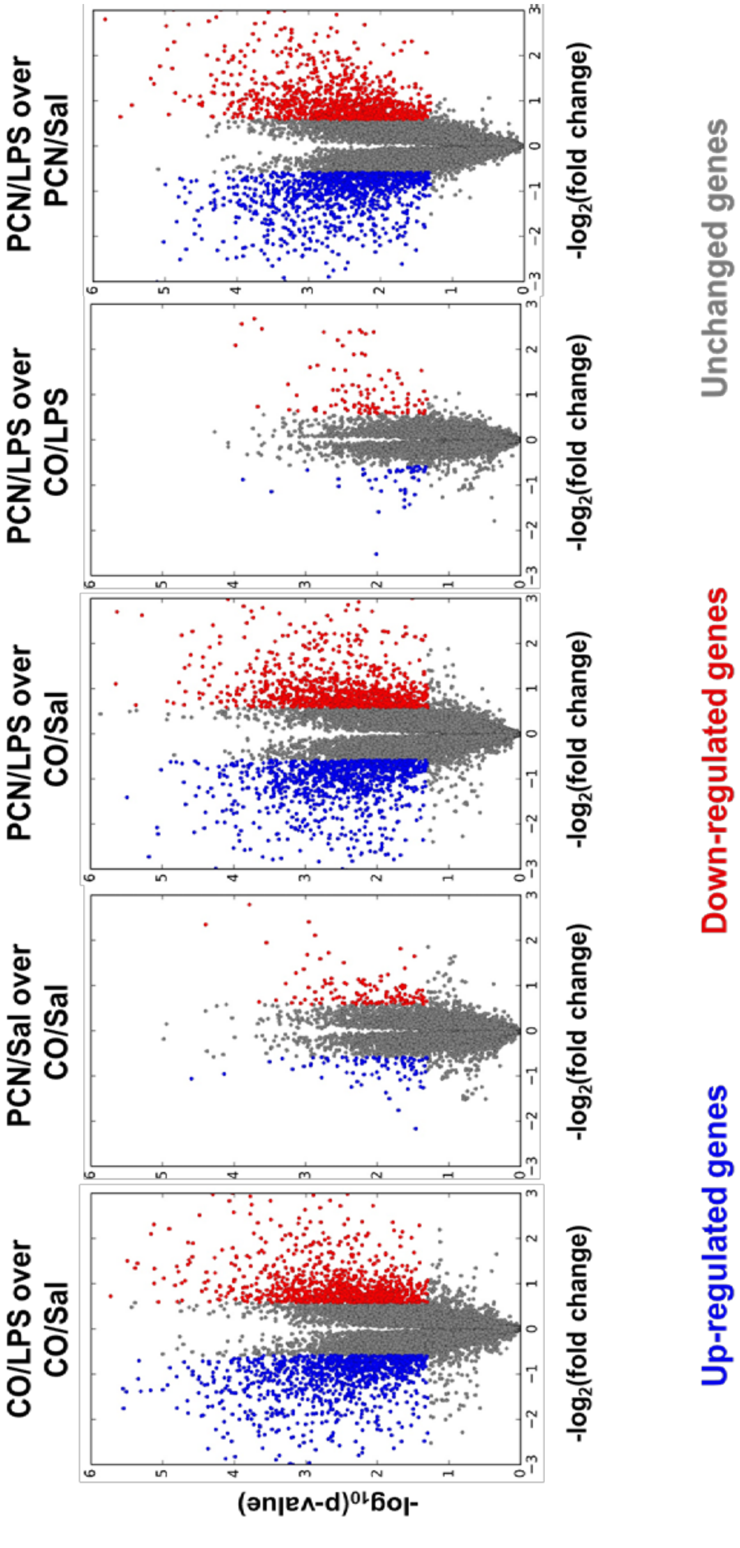

Supplementary Figure S2. Heatmap showing the differentially regulated genes with the combined PCN and LPS treatment compared to A. LPS alone and B. PCN alone.

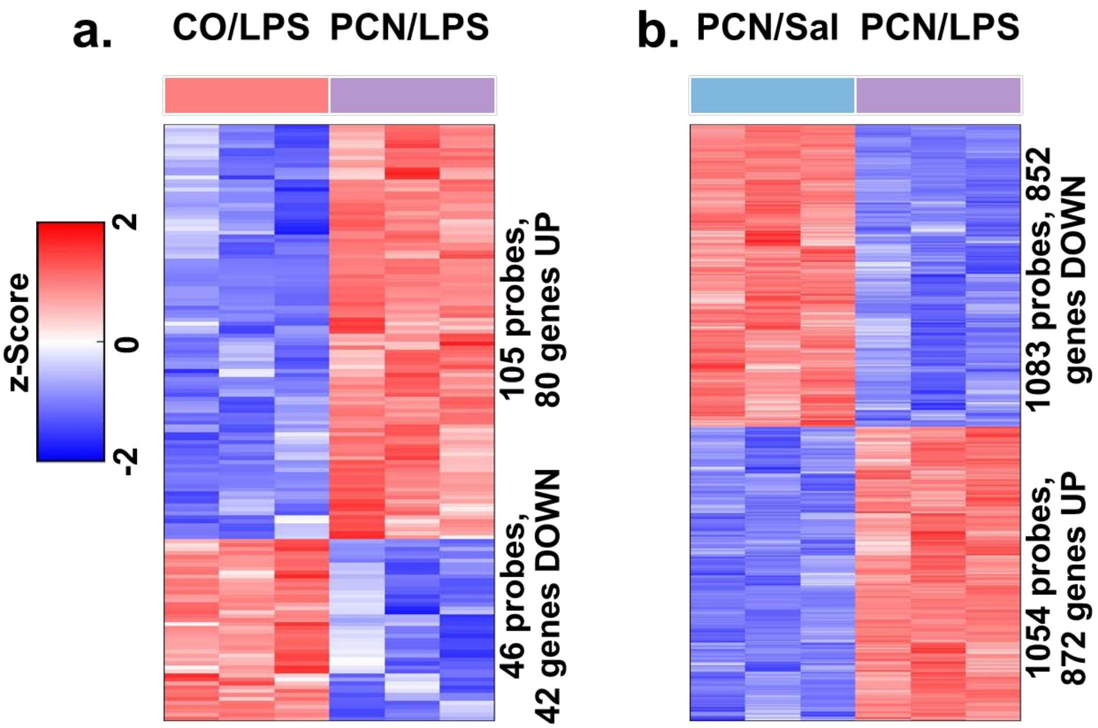

Supplementary Table S1. Fold changes in the gene expression of the top 15 genes with PCN and LPS treatment.

a. Significantly up-regulated genes ordered from highest to the lowest fold change w.r.t. PCN treatment.

	Fold Change		
**Gene Symbol**	**PCN vs control**	**LPS vs control**	**PCN/LPS vs control**
GSTM3	5.16	-1.59	1.54
CYP2C55	4.92	-1.14	1.24
CES6	3.88	-0.64	3.53
GSTA1	3.83	-2.24	
GSTA2	3.48	-2.48	
CYP3A11	2.41	-2.64	2.15
GSTM6	2.11	-2.89	-1.34
GSTM2	1.95	-1.87	
HSD17B6	1.72	-1.50	0.88
GSTM2	1.68	-1.22	0.66
CYP3A25	1.36	-2.40	0.94
GSTT3	1.13	-1.28	-0.90
EPHX1	1.12	-0.60	
GSTA4	1.20	-1.46	-0.78
INMT	1.05	-3.47	-2.24

b. Significantly down-regulated genes ordered from lowest to the highest fold change w.r.t. PCN treatment.

	Fold Change		
**Gene Symbol**	**PCN vs control**	**LPS vs control**	**PCN/LPS vs control**
SAA4	-0.92	2.68	2.26
2200001I15RIK	-0.85	1.05	0.89
LRG1	-0.74	1.45	1.50
LCAT	-0.67	0.83	0.86
LDHA	-0.64	1.15	1.12
IIGP2	-0.62	0.94	1.00
ACTB	-0.61	0.61	
IGTP	-0.59	1.05	0.98

Supplementary Table S2. Altered transcription factors predicted to have putative binding sites on the CYP3A4 promoter using TRANSFAC analysis.

**PCN↓ LPS↓**	**PCN↑ LPS↑**	**PCN↓ LPS↑**	**PCN↑ LPS↓**
SP1	NFE2	**GABP**	MEF2
FOXO4		CETS1P54	
MAZ		**PEA3**	
E12		**STAT1**	
FREAC2		**NFKB**	
NFAT		**IRF**	
LEF1		**MYCMAX**	
AP4		SP1	
MYC		AP1	
HNF3		**HSF**	
ETS2			
PAX4			
MYOD			
NFY			
CHX10			
ERR1			
